# Bruxism treatment outcomes: A systematic review and meta-analysis

**DOI:** 10.1097/MD.0000000000046247

**Published:** 2026-05-12

**Authors:** Hassan Ahmed Assiri, Lama Fahad Almuawi, Batool Abdullah Asiri, Shatha Tareq Abumelha, Raghad Musfer Alahmari, Mohammad Shahul Hameed, Sonia Egido-Moreno, Jose López-López

**Affiliations:** a Department of Diagnostic Science and Oral Biology and Periodontology, College of Dentistry, King Khalid University, Abha, Saudi Arabia; b Internship, College of Dentistry, King Khalid University, Saudi Arabia; c Department of Odontostomatology, Faculty of Medicine and Health Sciences, School of Dentistry, University Campus of Bellvitge, University of Barcelona, Barcelona Dental Hospital [HOUB], Barcelona, Spain.

**Keywords:** bruxism, occlusal splint, pharmacotherapy, therapy, treatment outcome

## Abstract

**Background:**

This review aimed at addressing the treatment outcomes of bruxism.

**Methods:**

The systematic review protocol was registered in International Prospective Register of Systematic Reviews with protocol number (CRD42024597809). It was conducted following the Preferred Reporting Items for Systematic Reviews and Meta-Analyses. The search was performed to retrieve the relevant articles from PubMed, Web of Science, and Scopus databases. The inclusion criteria were predefined as original studies including randomized and non-randomized controlled trials, case series, studies published between October 2019, and October 2024 published in English. The retrieved studies suitable for analysis were subjected for data extraction and risk of bias assessment using the Joanna Brigg Institute checklists for the relevant design of the studies.

**Results:**

Finally, 22 studies were suitable for the data extraction and risk of bias assessment. Studies revealed that occlusal splints, both conventional and 3-dimensional-printed, have been widely studied for their ability to reduce nighttime muscle activity. These devices help distribute occlusal forces and alleviate symptoms, although their effectiveness varies depending on design and material. Botulinum toxin type A has been examined as a treatment option, particularly for reducing pain and muscle activity in patients with bruxism. Biofeedback devices have also been explored as a noninvasive alternative to control bruxism.

**Conclusion:**

Overall, there is no single treatment that is universally effective for all patients. A multidisciplinary approach combining different therapies may yield the best results. Further long-term studies with rigorous methodological control are essential to evaluate the durability of therapeutic effects and determine the most cost-effective interventions.

## 1. Introduction

The phenomenon of bruxism can significantly influence oral health and affect the quality of patients’.^[1]^ Various definitions of bruxism have been proposed. In 2010, sleep bruxism (SB) was defined as stereotyped oromandibular activity during sleep, characterized by teeth grinding and clenching.^[2]^ In 2012, bruxism was described as the habit of grinding or clenching the teeth, either during sleep or unconsciously while awake.^[1]^ Subsequently, in 2013, an international consensus defined bruxism as a repetitive activity of the masticatory muscles, characterized by clenching or grinding of the teeth and/or by thrusting or clenching of the jaw. This condition is classified into SB and wake bruxism, based on its circadian manifestation.^[3]^ Bruxism causes can be central or peripheral. The peripheral factors include stress anxiety and genetic factors while central causes encompass disorders at the level of neurotransmitters and the basal ganglia, as well as organic and functional changes in the nervous system.^[4–6]^ Regarding psychological factors, some researchers assert that high levels of anxiety, stress, and emotional tension are the most prevalent causes. It has been reported that some types of physical training may contribute to bruxism occurrence.^[7]^ Furthermore, certain circumstances such as smoking, the use of specific medications, and respiratory problems can be considered risk factors for bruxism.^[8]^ There are 2 types of bruxism based on their etiology: primary or idiopathic bruxism, not associated with any disease, and secondary or iatrogenic bruxism, occurring in the presence of an underlying disease.^[9]^ Understanding the causal factors is essential for diagnosis and treatment planning. The available therapeutic options for managing the potentially detrimental effects of SB have varying degrees of effectiveness.^[10]^ Traditionally, dentists recommended occlusal adjustment, as occlusal imbalance was believed to be the primary cause of bruxism.^[11]^ Occlusal adjustments often involve the use of splints on the maxillary or mandibular arch to alleviate occlusal interferences, protect tooth surfaces, and relax masticatory muscles. However, there is no evidence to demonstrate that these measures prevent SB.^[12,13]^ Techniques such as biofeedback and relaxation have been suggested to be effective, particularly in daytime bruxism, which is more closely linked to stress.^[14,15]^ Experts also recommend sleep hygiene techniques, such as avoiding caffeine consumption and engaging in relaxation activities before bedtime, to control SB. However, recent research indicates that these therapies are ineffective in controlling muscle activity when the autonomic nervous system remains activated.^[16]^ Additionally, the use of portable devices with contingent electrical stimulation has been considered a promising approach for treating bruxism.^[16–18]^ Regarding pharmacological interventions, certain medications have shown the potential to reduce bruxism episodes. Injecting botulinum toxin (BTX) into the masticatory muscles is emerging as a promising option, as it decreases muscle activity in affected patients. However, the evidence is still insufficient to recommend it routinely, in addition to the potential adverse effects from its use.^[19]^ The long-term use of certain pharmaceutical therapies may raise concerns due to potential adverse effects or dependency issues.^[20]^ Furthermore, mandibular advancement devices have been shown to probably help reduce muscle activity in bruxism.^[21]^ Providing the various approaches and treatment being used to manage bruxism, this systematic review aims to summarize the evidence of bruxism treatment outcomes, and hence answer the following research question:

What are the treatment outcomes for patients with bruxism?

## 2. Data source

The systematic review protocol was registered and can be accessed in International Prospective Register of Systematic Reviews with protocol number (CRD42024597809). The review was conducted following the Preferred Reporting Items for Systematic Reviews and Meta-Analyses (PRISMA).^[22]^ The review question was formulated according to the Population or Problem, Intervention or Exposure, Comparison, Outcome framework. Quality assessment of the included studies was conducted using the Joanna Briggs Checklist tool.^[23]^ The research question was specified as follows: “What are the treatment outcomes for patients presenting with bruxism?” The Population or Problem, Intervention or Exposure, Comparison, Outcome elements were defined as follows:

P** =** Patients with bruxism.

I **= **Different management modalities such as occlusal splints and pharmacotherapy.

C** = **Comparison of 1 treatment with another treatment or program to control bruxism.

O** = **The outcome of the treatment or management being utilized.

### 2.1. Inclusion criteria

Original studies including randomized and non-randomized controlled trials.

*Case series*: Studies published between October 2019 and October 2024 that met the inclusion and exclusion criteria.

### 2.2. Exclusion criteria

Narrative reviews.
Systematic reviews.
Book chapters.
Case reports.

### 2.3. Search strategy

The search strategy was designed for the databases (PubMed, Scopus, and Web of Science), using keywords in October 2024. In PubMed, we searched using the following terms: (“treatment outcomes of bruxism*” OR “therapy outcomes of bruxism*” OR “Sleep bruxism*” OR “Bruxism therapy*”) on October 1st, 2024. In Scopus, we searched according to TITLE-ABS-KEY (bruxism) AND TITLE-ABSKEY (treatment) on October 1st. In the Web of Science, we searched using the following term combination: bruxism (word variations: ti, ab, kw) AND treatment outcomes (word variations: ti, ab, kw) AND therapy (word variations: ti, ab, kw) on October 1st. The search was performed and reported according to the PRISMA flowchart for systematic reviews.

### 2.4. Recovery of studies

Studies retrieved from the databases were subjected to duplicate checking using Rayyan platform. Then, 5 authors (H.A., L.A., B.A., S.A., R.A.) independently screened the results for title and abstract according to the inclusion and exclusion criteria. The included studies are further assigned for the full text screening by the 5 authors independently. In cases of uncertainty, discussions were held among the authors to reach a consensus on whether to include or exclude a study based on predefined criteria. All the excluded articles were clear that they did not meet the inclusion criteria.

### 2.5. Data collection

Data extraction was performed for the included articles based on a pre-identified working table (Table 1). The 5 authors divided the included articles among themselves for extraction. In cases of uncertainty, the authors were consulted for a unified agreement. The data extracted based on the author and year, title, study design, sample, study duration, study aim, and study outcomes (Table 1).

**Table 1:** Characteristics and data extraction of the selected studies.

Characteristics of the selected studies						
References	Title	Study design	Sample	Study duration	Study aim	Outcome
Cruse et al^[24]^	Efficacy of botulinum toxin type A in the targeted treatment of sleep bruxism: a randomized, double-blind, placebo-controlled crossover study.	Double-blind, randomized, placebo-controlled, crossover study.	41 participants recruited, 35 randomized, and data from 22 analyzed (14 women).	3–6 mo	To evaluate the efficacy and safety of BTX-A injection in the treatment of sleep bruxism, to determine the optimal treatment strategy, the most effective doses, the duration of benefit, and the factors associated with therapeutic response.	Compared to a placebo, botulinum toxin type A (BTX-A) injections effectively decreased objectively recorded bruxism occurrences using electromyography, especially at larger dosages and with additional muscle groups (medial pterygoid, temporalis, and masseter). However, subjective reductions in pain and symptoms did not reach statistical significance, and efficacy decreased 12 wk after injection, suggesting short-term efficacy.
Shehri et al^[25]^	Evaluating the efficacy of low-dose botulinum toxin injection into the masseter muscle for the treatment of nocturnal bruxism: A randomized controlled clinical trial.	Randomized controlled clinical trial with parallel group.	22 patients with nocturnal bruxism (11 in the Botox group and 11 in the placebo group).	6 mo–1 yr	To evaluate the efficacy of injecting 10 units of BTX-A into the masseter muscle to reduce nocturnal bruxism.	For around 3 mo, injections of 10 units of botulinum toxin type A (BTX-A) into the masseter muscle successfully reduced nocturnal bruxism-related muscular activity and related pain. However, symptoms showed a short-term efficacy and returned after this time.
Al-Wayli et al^[26]^	Treatment of chronic pain associated with nocturnal bruxism with botulinum toxin: A prospective and randomized clinical study.	Prospective, randomized, controlled clinical study with parallel groups.	50 female patients with nocturnal bruxism associated with chronic pain in the masseter muscles, with a mean age of 45.5 ± 10.8 yr.	6 mo–1 yr	To evaluate the effect of BTX-A in treating the pain associated with nocturnal bruxism, comparing it with traditional methods such as behavioral therapy, occlusal splints, and anti-inflammatory drugs.	In contrast to traditional methods, botulinum toxin type A injections effectively decreased pain during nocturnal bruxism by reducing masseter muscle activation. Improvement persisted during the 1-yr follow-up, suggesting that this therapy is efficacious over the long run.
da Silva Ramalho et al^[27]^	Effect of botulinum toxin A on pain, bite force, and satisfaction of patients with bruxism: A randomized, single-blind clinical trial comparing 2 protocols.	Randomized, 2-group, parallel, single-blind clinical trial (only patients were blinded).	20 participants, over 18 yr of age, with bruxism and bilateral orofacial pain of muscular origin, excluding those with TMD, allergies to BTX-A, or neuromuscular problems.	6 mo–1 yr	To evaluate bite force, orofacial pain perception, and treatment satisfaction in patients with bruxism using 2 BTX-A injection protocols.	The toxin botulinum A injections into the masseter muscle alone or the masseter and temporal muscles together dramatically decreased bite force and orofacial pain in bruxism patients, leading to excellent patient satisfaction. Particularly for short-term management up to 120 days, both injection techniques showed comparable efficacy.
Kaya & Ataoğlu^[28]^	Botulinum toxin treatment of temporomandibular joint pain in patients with bruxism: a prospective, randomized clinical study.	Prospective, randomized clinical study.	40 patients with bruxism, aged between 18 and 45 yr (mean 26.33), of whom 7 were men and 33 were women. The patients were randomly assigned to 2 groups: 1 treated with botulinum toxin in the masseter muscles and the other with occlusal splints.	6 mo–1 yr.	To compare the efficacy of occlusal splints with botulinum toxin administration for the treatment of temporomandibular joint pain in patients with bruxism.	Botulinum toxin (BTX-A) injections and occlusal splint therapy both decreased bruxism-related pain, however neither method was better than the other. With occlusal splints, biting force stayed steady but increased at 6 mo, but maximum bite force briefly decreased following BTX-A injections and returned to normal at 3 mo.
Shim et al^[29]^	Botulinum toxin therapy for managing sleep bruxism: A randomized and placebo-controlled trial.	Randomized placebo-controlled clinical trial.	30 participants with SB, of whom 23 completed the study (10 in the placebo group and 13 in the treatment group).	6 mo–1 yr	To evaluate the effects of BTX-A for the management of sleep bruxism using polysomnography recordings in a randomized placebo-controlled trial.	For up to 12 wk, a single dose of botulinum toxin type A reduced the contraction force of the masseter muscle during sleep bruxism episodes, but not the frequency or overall occurrence of rhythmic masticatory muscle activity. Therefore, by decreasing muscular force but leaving bruxism occurrences unabated, botulinum toxin type A injection effectively cures sleep bruxism.
Hosgor et al^[30]^	Comparison of the efficacy of occlusal splint and botulinum toxin therapies in patients with temporomandibular disorders with sleep bruxism.	Retrospective cohort study.	60 patients (49 women and 11 men, mean age 34.63 ± 11.85 yr), divided into 2 groups of 30 patients each (occlusal splint group and botulinum toxin group).	6 mo–1 yr.	To evaluate the efficacy of occlusal splint and botulinum toxin in reducing pain and improving the mouth opening in patients with TMD and sleep bruxism.	In individuals with temporomandibular disorders and sleep bruxism, botulinum toxin (BTX) and occlusal splint therapy reduced pain and increased mouth opening. However, BTX reduced symptoms earlier with initial treatment, making it a better first treatment for those with severe discomfort.
Rabel et al^[31]^	3D-printed versus milled stabilization splints for the management of bruxism and temporomandibular disorders: study protocol for a prospective randomized single-blinded crossover trial.	Prospective, randomized, crossover, single-blind, 2-cohort clinical trial.	40 participants, divided into 2 cohorts: 20 with bruxism and 20 with pain-related temporomandibular disorders (TMD) (myalgia, myofascial pain, or arthralgia of the jaw muscles/temporomandibular joints), all treated at the Department of Prosthetic Dentistry, University of Freiburg, Germany.	1 yr (prospective study)	To compare the noninferiority of 3D-printed versus milled stabilizing splints in terms of oral health-related quality of life, therapeutic efficacy (reduced pain and tooth wear), and technical outcomes (fit, wear, and fracture rate) in patients with bruxism or TMD.	For bruxism and temporomandibular disorders (TMD), 3-dimensionally printed stabilizing splints can produce comparable clinical results as milled splints. The trial will conclude that 3D printed splints are not less effective in terms of splint durability, treatment efficacy, or patient quality of life.
Wang et al^[32]^	Preliminary clinical evaluation of traditional and new digital PEEK occlusal splints for the management of sleep bruxism.	Preliminary comparative clinical study.	16 individuals with a clinical diagnosis of sleep bruxism, aged between 18 and 44 yr. They were randomly divided into 2 groups of 8 people: 1 group with digital splints (test group) and another with traditional splints (control group).	6 mo–1 yr	To compare manual time and preliminary clinical effects between digital and traditional occlusal splints for treating patients with sleep bruxism.	When compared to acrylic resin splints, digitally produced PEEK occlusal splints demonstrated significantly better comfort, greater wear resistance, and increased time efficiency for treating sleep bruxism. However, the retention of both splint types was comparable, which was clinically acceptable.
Damar et al^[33]^	A comparison of manual therapy and splint therapy in patients diagnosed with myofascial temporomandibular dysfunction with sleep bruxism.	Randomized controlled clinical trial.	29 women aged 18 to 50 yr diagnosed with myofascial temporomandibular dysfunction and SB, divided into 2 groups: - Manual therapy group: 15 patients. - Splint therapy group: 14 patients.	6 mo–1 yr.	To evaluate the effect of manual therapy on temporomandibular pain, range of motion, jaw function, sleep quality, and patient satisfaction.	Both manual and splint therapy improved sleep, function, pain, and range of motion in the temporomandibular joint (TMJ) in individuals with myofascial temporomandibular dysfunction associated with sleep bruxism. But compared to splint therapy, manual therapy significantly improved these metrics and patient satisfaction.
Bargellini et al^[34]^	Short-term effects of 3D-printed occlusal splints and conventional splints on sleep bruxism activity: EMG–ECG night recordings of a sample of young adults.	Randomized clinical trial.	26 participants (19 men and 7 women) with an average age of 25.8 ± 2.6 yr.	Approximately 6 mo.	To compare the effects of 3D-printed splints and conventionally manufactured splints on EMG activity in sleep bruxism (SB).	The 3D splint reduces the rhythmic muscle activity and patients comfort enhanced compared to conventional one.
Bergmann et al^[35]^	Effect of treatment with a full-occlusion biofeedback splint on sleep bruxism and TMD pain: a randomized controlled clinical trial.	Randomized controlled clinical trial.	Included 41 patients who were randomly assigned to 2 groups: •Test group: 10 women, 9 men •Control group: 11 women, 9 men	6 mo–1 yr.	The purpose of the study was to analyze the outcome of treatment with a complete occlusion biofeedback splint on SB and TMD pain compared to treatment with a close-fitting occlusal splint.	When compared to an adjusted occlusal splint, a full-occlusion biofeedback (BFB) splint reduced the frequency and length of sleep bruxism attacks, as well as the discomfort felt by the face muscles and overall. The biofeedback splint’s effectiveness in controlling sleep bruxism is demonstrated by the fact that its positive effects persisted even after treatment ended.
Ali et al^[36]^	Botulinum toxin and occlusal splints for the management of sleep bruxism in individuals with implant overdentures: A randomized controlled trial	Single-blind, randomized clinical trial with a control group, pretest and post-test	42 patients	1 yr	To evaluate the efficacy of occlusal protection and botox injections in the treatment of SB in subjects whose edentulous arches had been restored with implant-supported OD.	When compared to control patients who slept with their overdentures, patients with implant-supported overdentures reported significantly higher levels of patient satisfaction and better quality sleep after receiving botulinum toxin (BTX) injections and occlusal splints. At the 12-mo follow-up, the BTX group also showed better results in terms of patient satisfaction, sleep quality, and less prosthodontic (mechanical) issues.
Haggiag et al^[37]^	A novel biofeedback approach for the control of awake bruxism and chronic migraine: use of a posterior interocclusal appliance in awake patients.	Interventional, prospective, non-randomized, self-controlled clinical trial.	74 patients with chronic migraine and nighttime bruxism, selected from a total of 223 patients with orofacial pain in a dental office.	3–6 mo	To evaluate pain reduction in patients with chronic migraine and awake bruxism by using a biofeedback-based partial posterior interocclusal appliance.	Within the first 30 days, the use of a posterior interocclusal device intended for biofeedback treatment of awake bruxism effectively decreased pain, including chronic migraine. The improvement persisted for up to a year, despite the device being discontinued after 90 days, indicating efficient and durable awake bruxism and pain management.
Pfeiffer et al^[38]^	Is biofeedback through an intra-aural device an effective method to treat bruxism? Case series and initial experience.	Prospective controlled case series study.	Seven female patients with a mean age of 47.3 yr (range 23–64 yr).	6 mo–1 yr	To evaluate the efficacy of a biofeedback-based intra-aural device for bruxism treatment and to provide the first clinical evidence of its impact on symptom reduction.	By raising patient awareness of the habit, biofeedback using an intra-aural device demonstrated promise in lowering bruxism-related headache and pain sensations. Although the early results were positive, some patients had pain and fit problems, necessitating long-term testing and device modification to verify efficacy.
Ohara et al^[39]^	Effects of vibratory feedback stimuli through an oral appliance on sleep bruxism: a 6-wk intervention trial.	Prospective, single-arm, open-label, interventional study.	10 participants diagnosed with “definitive” sleep bruxism.	6 wk	To evaluate the effect of vibratory stimulation via an oral appliance on masticatory muscle activity related to sleep bruxism when applied for 4 wk after a 2-wk adaptation period.	Over the course of 4 wk, a vibratory feedback stimulus delivered by a dental appliance effectively reduced the frequency and duration of sleep bruxism. However, the effects vanished as soon as the stimulus was stopped, indicating that they cannot be sustained unless they are utilized continuously.
Klitynska et al^[40]^	Effectiveness of bruxism treatment in young adults.	Cohort study.	377 individuals aged 25 to 44 yr.	6 mo–1 yr	To evaluate the effectiveness of a treatment algorithm and preventive measures aimed at eliminating the clinical manifestations of bruxism in young people.	After 1-yr, selective grinding in orthodontic patients significantly reduced bruxism. In TMJ patients, significant improvement observed. In psychoemtional patients, also significant reduced of bruxism observed
Mostafavi et al^[41]^	The efficacy of low and moderate dosage of diazepam on sleep bruxism in children: A randomized placebo-controlled clinical trial	Randomized controlled clinical trial.	90 individuals aged 2 to 8 yr	6 mo–1 yr	To assess the effect and safety of a short course of diazepam to control bruxism in healthy children.	Low- or moderate-dose diazepam for a short duration had no notable effect on sleep bruxism versus placebo. Additionally, diazepam had a higher rate of drowsiness on the next day, with potential for limited clinical usefulness and possible side effects.
Wieckiewicz et al^[42]^	Consecutive controlled case series on effectiveness of opipramol in severe sleep bruxism management – Preliminary Study on New Therapeutic Path.	Consecutive case-controlled series study.	19 healthy participants with severe bruxism, diagnosed by phase I video polysomnography. The sample included 14 women and 5 men, aged 20 to 47 yr (mean: 32.32 ± 8.12).	6 mo–1 yr	To preliminarily determine the effectiveness of opipramol in managing severe sleep bruxism.	Patients who took 100 mg before bed experienced a 79% decrease in severe sleep bruxism attacks, and both phasic and tonic muscular activity were significantly reduced. Opipramol treatment showed promise as a successful treatment for severe sleep bruxism because it was well tolerated and had no discernible effects on general sleep metrics.
Kobayashi, et al^[43]^	Immediate evaluation of the effect of photo biomodulation with infrared LEDs on childhood sleep bruxism: a randomized clinical trial	Randomized clinical trial (randomized clinical trial)	Thirty children (n = 30), divided into 3 groups: •Group 1: control/absence of bruxism (n = 10) •Group 2: bruxism treated with infrared LED (n = 10) •Group 3: bruxism treated with occlusal splint (n = 10)	3–6 mo	To evaluate the effects of infrared LED photo biomodulation therapy in children with SB.	In children with sleep bruxism, infrared LED photobiomodulation therapy did not significantly affect salivary dopamine levels or electromyographic masticatory muscle activity. In contrast to controls, occlusal splint therapy demonstrated elevated salivary dopamine levels prior to treatment and enhanced masticatory muscle activity at rest.
Mousa et al^[44]^	Effect of different treatment modalities on masseter inhibitory reflex in patients with sleep bruxism: A case–control study.	Case-control study.	100 individuals, divided into 4 groups of 25: G1: Conservative treatment. G2: Treatment with occlusal splint. G3: Low-level laser therapy. G4: Control group (without treatment).	6 mo–1 yr	To investigate changes in the masseter inhibitory reflex in patients with sleep bruxism and to evaluate the effect of different treatment modalities on the reflex.	Among all forms of conservative therapy and occlusal splints, low-level laser therapy (LLLT) demonstrated to be the best effective treatment for patients with sleep bruxism who displayed aberrant masseter inhibitory reflex (MIR). After a month of treatment, LLLT was able to successfully change the MIR parameters by increasing length and decreasing latency.
Crăciun et al^[45]^	Study on the monitoring of nocturnal bruxism in children and adolescents using the Bruxoff device.	Prospective study conducted over 6 mo (March–September 2022).	51 participants (36 with bruxism and 15 without bruxism), between 5 and 18 yr old.	6 mo–1 yr	*Bruxoff* device in the monitoring and early diagnosis of nocturnal bruxism in children and adolescents, analyzing the intensity and duration of parafunctional activity.	The study showed that by accurately measuring heart rate and masseter muscle activity, the Bruxoff device can be used to screen for and diagnose nocturnal bruxism in children and adolescents at an early stage. Statistically significant differences between the control and bruxism groups demonstrated clinical use for screening and evaluating the severity of bruxism.

BTX = botulinum toxin, LLL = low-level laser therapy, MI = masseter inhibitory reflex, SB = sleep bruxism.

### 2.6. Risk of bias assessment

Each study was assessed using the checklist recommended for its specific study type. The Joanna Briggs Institute (JBI) critical appraisal checklist was applied to each study design used.^[46]^ To minimize biased assessment, all 5 authors independently reviewed all studies. The rating system was established before the critical appraisal began to standardize the bias assessment criteria. The risk of bias is considered low quality if <40% of the checklist is met, average quality if between 40% and 80% is met, or high quality if more than 80% is met.^[47]^

## 3. Results

### 3.1. Selection of studies

**Figure 1.**
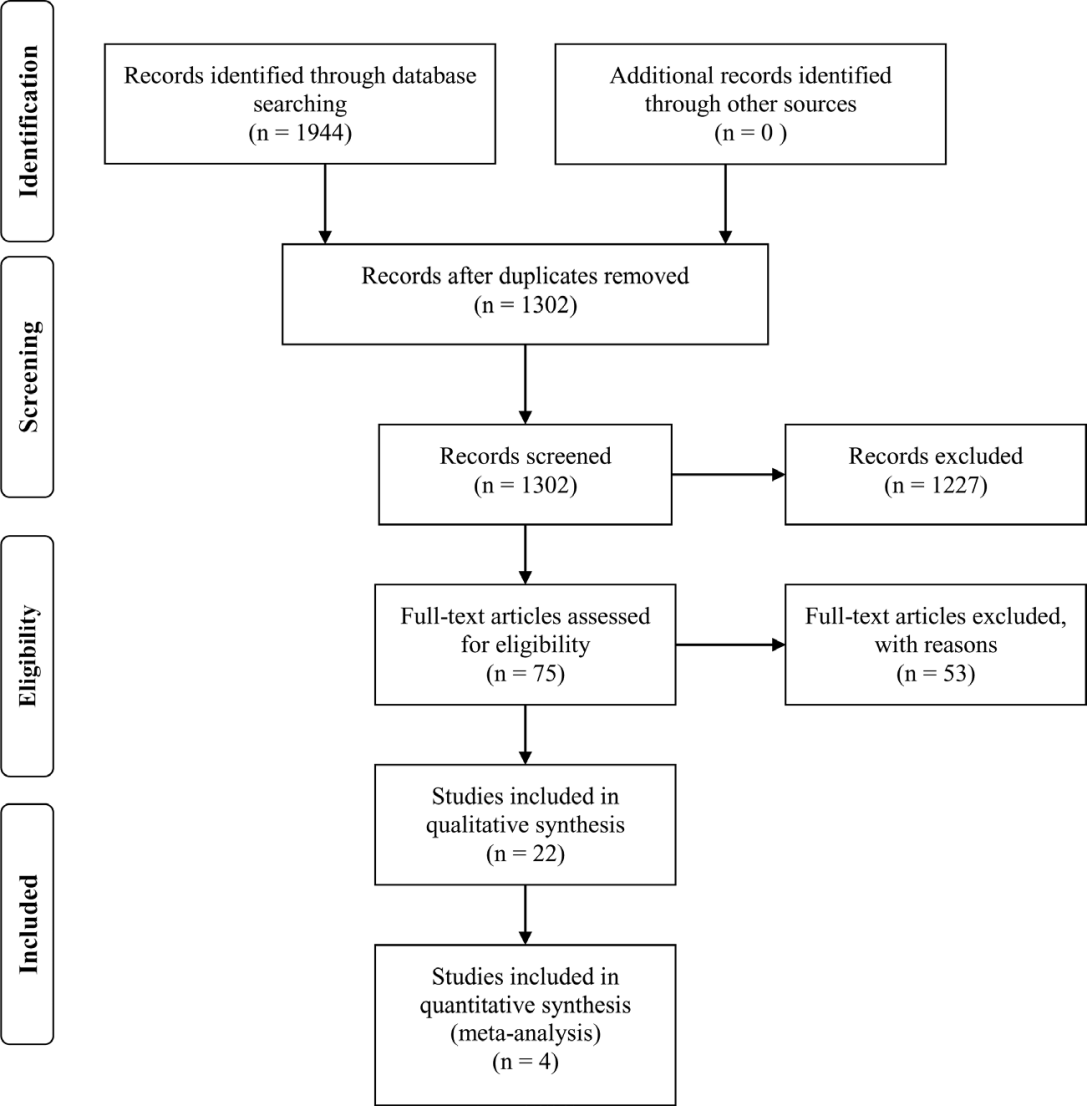
PRISMA flowchart of conducting systematic reviewes.

The search of the 3 databases yielded 1944 articles: 590 from PubMed, 602 from Scopus, and 752 from Web of Science. After the process of duplicate check, title and abstract screening, full text screening, 22 studies are deemed suitable for data extraction and quality assessment.^[24–45]^ The PRISMA flowchart illustrates the process of study retrieval (Fig. 1).

### 3.2. Risk of bias assessment

The risk of bias assessment is conducted for the final relevant studies and is classified as low, moderate, or high, based on the following criteria including proper randomization, blinding of the evaluator and participants, adequate control group, sufficient follow-up, appropriate statistical analysis and clear description of the sample and inclusion criteria. Risk of bias assessment of randomized clinical trials (Table 2). Risk of bias assessment of observational studies and case series (Table 3). Risk of bias assessment of cohort studies (Table 4).

**Table 2:** Bias assessment of randomized clinical trials (RCTs).

Study title	Year	Randomization	Blinding	Control group	Follow-up	Statistical analysis	Bias level	Comments
Evaluation of the efficacy of low-dose botulinum toxin injection into the masseter muscle for the treatment of nocturnal bruxism: A randomized controlled clinical trial.^[25]^	2022	Yes	Double-blind	Yes	Medium-term	Adequate	Low	Robust clinical trial with appropriate randomization and blinding; follow-up was sufficient to evaluate medium-term efficacy.
Treatment of chronic pain associated with nocturnal bruxism with botulinum toxin: A prospective and randomized clinical study.^[26]^	2017	Yes	Not reported	Yes	Short-term	Adequate	Moderate	Although randomization and a control group were used, the absence of blinding information may introduce bias.
Effect of botulinum toxin A on pain, bite force, and satisfaction of patients with bruxism: A randomized, single-blind clinical trial comparing 2 protocols.^[27]^	2023	Yes	Single-blind	Yes	Short-term	Adequate	Moderate	Lack of full blinding and long-term follow-up
Botulinum toxin treatment of temporomandibular joint pain in patients with bruxism: A prospective, randomized clinical study.^[28]^	2021	Yes	Not reported	Yes	Short-term	Adequate	Moderate	Lack of information on blinding and short follow-up duration may affect the study’s internal validity.
Botulinum toxin therapy for managing sleep bruxism: A randomized and placebo-controlled trial.^[29]^	2020	Yes	Double-blind	Yes	Short-term	Adequate	Low	Strong design with randomization and double blinding; however, short follow-up may not capture long-term effects.
Efficacy of botulinum toxin type A in the targeted treatment of sleep bruxism: a randomized, double-blind, placebo-controlled crossover study.^[30]^	2022	Yes	Double-blind	Yes	Adequate	Robust	Low	Solid methodology with proper control
A comparison of manual therapy and splint therapy in patients diagnosed with myofascial temporomandibular dysfunction with sleep bruxism.^[33]^	2022	Yes	Not reported	Yes	Short-term	Adequate	Moderate	Although randomization and a control group are mentioned, the lack of information on blinding may introduce performance and detection biases.
Short-term effects of 3D-printed occlusal splints and conventional splints on sleep bruxism activity: EMG–ECG night recordings of a sample of young adults.^[34]^	2024	Yes	Single-blind	Yes	Short-term	Adequate	Moderate	Well-designed but limited follow-up
Effect of treatment with a full-occlusion biofeedback splint on sleep bruxism and TMD pain: a randomized controlled clinical trial.^[35]^	2020	Yes	Not reported	Yes	Not reported	Limited	Moderate	Unclear methodology regarding follow-up
Botulinum toxin and occlusal splints for the management of sleep bruxism in individuals with implant overdentures: A randomized controlled trial.^[36]^	2021	Yes	Not reported	Yes	Not reported	Adequate	Moderate	Lacks information on randomization and blinding
The efficacy of low and moderate dosage of diazepam on sleep bruxism in children: A randomized placebo-controlled clinical trial.^[41]^	2019	Yes	Double-blind	Yes	Short-term	Adequate	Low	Well-structured study with randomization and double blinding; short follow-up is a limitation for assessing long-term effects.
Immediate evaluation of the effect of infrared LED photobiomodulation on childhood sleep bruxism: a randomized clinical trial.^[43]^	2022	Yes	Double-blind	Yes	Short-term	Adequate	Low	Well-designed study with randomization and double blinding; however, the short follow-up limits the assessment of long-term effects.

**Table 3:** Bias risk assessment in observational studies and case series.

Study title	Year	Participant selection	Evaluator blinding	Comparability	Statistical analysis	Bias level	Comments
Preliminary clinical evaluation of traditional and new digital PEEK occlusal splints for the management of sleep bruxism.^[32]^	2020	Convenience	No	No	Descriptive	High	Convenience sampling, lack of blinding, and absence of comparability reduce the validity of the findings.
A new biofeedback approach for awake bruxism and chronic migraine: using a posterior interocclusal device.^[37]^	2019	Convenience	No	No	Descriptive	High	Lack of control and randomization, along with convenience sampling, increase the risk of bias.
Is biofeedback through an intra-aural device an effective method to treat bruxism? Case series and initial experience.^[38]^	2020	Convenience	No	No	Descriptive	High	Lack of control and randomization.
Effects of vibratory feedback stimuli through an oral appliance on sleep bruxism: a 6-wk intervention trial.^[39]^	2021	Convenience	Not reported	Partial	Descriptive	Moderate	Preliminary design without a control group.
Consecutive controlled case series on effectiveness of opipramol in severe sleep bruxism management – Preliminary Study on New Therapeutic Path.^[42]^	2020	Convenience	No	Limited	Limited	High	No blinding or robust comparison.
Effect of different treatment modalities on masseter inhibitory reflex in sleep bruxism patients: A case–control study.^[44]^	2024	Convenience	No	No	Descriptive	High	The study investigates the effect of different treatment modalities on the masseter inhibitory reflex in sleep bruxism patients. However, insufficient information on blinding, participant selection criteria, and group comparability increases the risk of bias.

PEEK = polyether ether ketone.

**Table 4:** Bias risk assessment of cohort studies.

Study	Year	Cohort selection	Blinding	Follow-up duration	Statistical analysis	Bias level	Comments
Comparison of the efficacy of occlusal splint therapy and botulinum toxin in patients with temporomandibular disorders with sleep bruxism.^[30]^	2023	Convenience sampling	Not reported	Partial	Not reported	Moderate	The study compares 2 active treatments without clear randomization or blinding, leading to potential selection and detection biases. Additionally, the lack of detailed statistical methods raises concerns about the robustness of results.
3D printed versus milled stabilization splints for the management of bruxism and temporomandibular disorders: study protocol for a prospective randomized single-blinded crossover trial.^[31]^	2024	Randomized	Not reported	Medium-term	Robust	Moderate	Missing information on evaluator blinding.
Effectiveness of bruxism treatment in young adults.^[40]^	2024	Adequate	Not reported	Short-term	Adequate	Moderate	The study aimed to assess the effectiveness of a developed algorithm for treatment and prevention measures targeting bruxism in young individuals. Specific details regarding blinding, follow-up duration, and statistical methods were not provided in the available summary.
Study regarding the monitoring of nocturnal bruxism in children and adolescents using bruxoff device.^[45]^	2023	Adequate	Not reported	Short-term	Adequate	Moderate	Lack of blinding and long-term follow-up.

### 3.3. Statistical analysis and data synthesis

**Figure 2.**
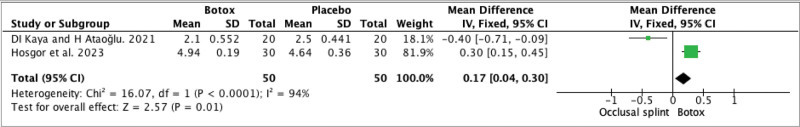
Meta-analysis of the studies comparing occlusal splints versus botulinum injection.

**Figure 3.**
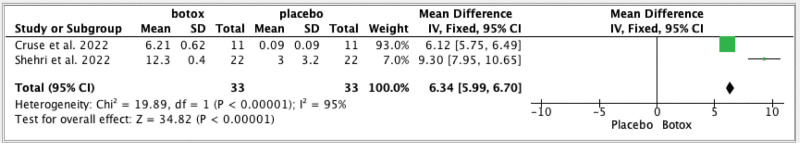
Meta-analysis of the studies comparing placebo protocol versus botulinum injection.

We identified 4 studies relevant for meta-analysis (Figs. 2 and 3). Accordingly, pooled analyses were performed using a random-effects model. Heterogeneity among the studies was assessed using the *I*^2^ statistics. Heterogeneity was considered statistically significant for a *P*-value < .05 and was interpreted as recommended by the Cochrane Handbook: 0% to 40% was considered unimportant, 30% to 60% as moderate heterogeneity, 50% to 90% as substantial heterogeneity, and 75% to 100% as considerable heterogeneity. The Review Manager (RevMan) version 5.4 (The Cochrane Collaboration, Copenhagen, Denmark) program was utilized to analyze the data, previously recorded in an Excel table. Forest plots were performed to graphically represent the differences in pain between various treatments, with a 95% confidence interval (CI).

The difference was statistically significant, favoring BTX treatment over occlusal splints (weighted mean difference: 0.17; 95% CI: 0.04–0.30, *P* = .01 and *I*² heterogeneity: 94%, *P* < .0001).

The difference was statistically significant, favoring BTX treatment over placebo (weighted mean difference: 6.34; 95% CI: 5.99–6.70, *P* < .00001 and *I*² heterogeneity: 95%, *P* < .0001).

## 4. Discussion

By comparing the results obtained from different investigations, similarities and discrepancies can be identified regarding the effectiveness of these approaches. In relation to the BTX as a treatment for bruxism, studies have evaluated the use of BTX type A in patients with sleep–awake bruxism. Cruse et al^[24]^ and Shehri et al^[25]^ found that injecting BTX into the masseter muscle significantly reduces muscle activity and bruxism symptoms. Similarly, Al-Wayli et al^[26]^ demonstrated that this therapy effectively reduces chronic pain associated with nocturnal bruxism. However, da Silva Ramalho et al^[27]^ compared 2 different BTX protocols and found variations in pain reduction and bite force, suggesting that effectiveness may depend on the dosage used. Kaya and Ataoğlu^[28]^ reported improvements in temporomandibular joint pain in patients with bruxism, although they did not detail its impact on bruxing activity specifically. In contrast, Shim et al^[29]^ highlighted that while BTX is effective in reducing pain and muscle hypertrophy, its impact on bruxism frequency remains uncertain. Furthermore, Hosgor et al^[30]^ compared BTX with occlusal splints and concluded that both therapies present similar benefits in managing bruxism, albeit through different mechanisms of action. Occlusal splint therapy has been extensively studied. Hosgor et al^[30]^ and Rabel et al^[31]^ compared different types of splints and found that both 3-dimensional (3D)-printed and milled splints can alleviate symptoms of bruxism and temporomandibular disorders. Wang et al^[32]^ evaluated a new polyether ether ketone splint and found it to perform comparably to traditional splints. It agrees with Damar et al^[33]^ who compared manual therapy with splint therapy and found that both strategies positively impacted pain reduction and functional improvement. Bargellini et al^[34]^ found that both splint types (3D-printed and conventional) effectively reduced nocturnal bruxing activity, although no significant differences were observed between the 2 devices. Bergmann et al^[35]^ demonstrated that biofeedback via an occlusal splint with electronic feedback can reduce bruxing activity and pain associated with tempromandinular joint disorder (TMD), suggesting a promising therapeutic option. In the study by Ali et al,^[36]^ BTX was shown to positively affect pain and muscle activity reduction, while occlusal splints also exhibited benefits but with slightly lower efficacy in reducing bruxing activity. The use of biofeedback and electronic devices have been investigated as a noninvasive alternative. Haggiag et al^[37]^ explored a posterior interocclusal device that showed promising results in reducing bruxism in awake patients. Pfeiffer et al^[38]^ evaluated an intra-aural device for biofeedback and found that it may be effective, although further longitudinal studies are needed. Ohara et al^[39]^ tested a vibratory feedback device and found that its use for 6 weeks reduced bruxing activity in some patients. Similarly, Klitynska et al^[40]^ reported improvements with electronic devices in young adults, although not all participants experienced significant reductions in bruxing frequency. The pharmacological treatment of bruxism has also been evaluated. Mostafavi et al^[41]^ studied the use of diazepam in children with bruxism and found that low and moderate doses can reduce nocturnal bruxing activity. Wieckiewicz et al^[42]^ investigated opipramol in severe cases of SB and found that it is potentially be a promising therapeutic option, although further studies are required. Considering possible innovative approach bruxism management, Kobayashi et al^[43]^ explored the effect of photobiomodulation with infrared LEDs in children with SB and reported immediate improvements in symptoms. Mousa et al^[44]^ investigated different therapeutic approaches in the masseter inhibitory reflex, finding differences in response depending on the modality used.

### 4.1. Study limitations

It is important to consider the various limitations of the studies that were part of this systematic review when evaluating the findings. The wide range of study types, including case–control studies, randomized controlled trials, and case series, makes comparison and generalizability impossible. Assessment of long-term efficacy and safety outcomes is limited by short follow-up periods, often ranging from 6 months to a year. In addition, heterogeneity in patient populations, therapies, and measuring tools is indicated by high statistical heterogeneity (*I*^2^ = 94–95%). Furthermore, the statistical power and precision of estimated treatment effects are diminished by the relatively small number of individuals in several research. Overall, the limitations encourage critical evaluation of the data that is now available and offer guidance for future research development.

## 5. Conclusion

The reviewed studies indicate no universally superior treatment for bruxism; rather. BTX has been shown to effectively reduce pain and muscle activity, although its impact on bruxism frequency remains inconclusive. Occlusal splints continue to be a widely utilized therapeutic option, while biofeedback devices and pharmacological therapies have yielded mixed results. Future research should focus on combined treatment protocols and long-term studies to determine the durability of therapeutic effects across different populations. Additionally, it is crucial to evaluate the cost-benefit ratio of each treatment, as well as potential long-term adverse effects. A multidisciplinary tailored approach and combination of therapies may represent the most effective strategy to address the various manifestations of bruxism and enhance patients’ quality of life.

## Acknowledgments

The authors acknowledge King Khalid University library for granting access to the data bases.

## Author contributions

**Data curation**: Lama Fahad Almuawi.

**Methodology**: Batool Abdullah Asiri.

**Supervision**: Mohammad Shahul Hameed.

**Validation**: Sonia Egido-Moreno, Jose López-López.

**Visualization**: Shatha Tareq Abumelha.

**Writing – original draft**: Raghad Musfer Alahmari.

**Writing – review & editing**: Hassan Ahmed Assiri.
